# Integrating DSM/ICD, Research Domain Criteria, and Descriptive Psychopathology in Teaching and Practice of Psychiatry

**DOI:** 10.3389/fpsyt.2018.00484

**Published:** 2018-10-05

**Authors:** Dimy Fluyau

**Affiliations:** Brain Health, Department of Psychiatry and Behavioral Sciences, Emory University School of Medicine, Atlanta, GA, United States

**Keywords:** DSM, ICD, psychopathology, RDoC, comparison, strengths and weaknesses, teaching and practice

## Abstract

**Background:** Some believe that Psychiatry relies solely on the Diagnostic and Statistical Manual of Mental Disorders (DSM). Some are not aware of the effort initiated by the Research Domain Criteria (RDoC) to propel the field to a new era of Medicine. Others are not acquainted with studies of Descriptive Psychopathology that can dissect symptoms and signs of mental illness and convert them into reliable clinical data for diagnosis and treatment purpose. This document is to bring keenness of the advances in research, translational or clinical, made in Psychiatry, and to encourage students, psychiatric residents, as well as psychiatric practitioners to integrate DSM/ICD, RDoC, and Descriptive Psychopathology into teaching and practice.

**Methods:** A search of the literature originated from 1985 to 2018 on two central databases: Google Scholar and Pubmed by free-texting: “comparison, strengths and weaknesses, Diagnostic and Statistical Manual of Mental Disorders, Research Domain Criteria, and Descriptive Psychopathology.”

**Results:** The DSM and ICD possess algorithms for psychiatric diagnosis, but they are limited to determine psychobiological causes of mental illnesses. Descriptive Psychopathology aims to dissect the mind to understand better “signs and symptoms,” their psychological, neurological, or neuropsychological origins but has been criticized for being non-reliable to practically explain the meaning of signs and symptoms that it attempts to describe. The RDoC claims to be a data-driven system of biological and psychological research for an evidence-based approach to Psychiatry. It is said that RDoC utilizes translational research that has been very slow to move to human experimentation.

**Discussion and conclusion:** Despite incommensurable translational research and human trials, the integration of translational research (neurosciences, experimental psychology, and genomics) as available human research data into teaching and practice is lacking. The author believes that the integration will enhance scientific and well-founded communication with our peers, advance psychopharmacologic treatments and improve our patient's mental well-being.

## Introduction

A 45-year-old man presented to the emergency on a rainy Saturday night complaining of suicidal ideation. The patient had been feeling down and drained for 3 weeks since his spouse divorced him. He had lost interest in joy, complained of fragmented sleep, frequent awakenings, and poor appetite. He felt like lifting 500 pounds of suffering while walking: “I am not myself, and I am already dead. The world is inexistent…, someone else replaces me.”

During a teaching session, the author asked a mentee to describe the symptoms that the patient experienced. The reply was that the patient reported symptoms of depression, but not all the criteria were met for a major depressive disorder. The author then added: “What did the patient mean when he said he had lost interest in joy?” “I believe it is called anhedonia.” The author then continued to ask: “Can you help to elucidate the psychopathology of anhedonia?” “I guess that anhedonia is a symptom of depression.” The author added: “You suggested the patient might have depression, can you describe some possible mechanisms of depression, in term of genetics, imaging, biochemical, and physiological changes.”

Some see Psychiatry as the practice of Medicine relying blindly on the Diagnostic and Statistical Manual of Mental Disorders (DSM) and has no association with any biological mechanisms of disease. One can argue that studies of Descriptive Psychopathology may provide appropriate tools to Psychiatrists to be able to understand and interpret symptoms and signs of mental illness and dissect them into reliable clinical data and to hypothesize on diagnosis and treatment. There is a need to treat and diagnose mental illnesses based on research combining psychology and neurobiology. RDoC seems to be the avant-garde.

Few university residency programs in the United States of America fully integrate knowledge of DSM/ICD, RDoc, or Descriptive Psychopathology in their core curricula. This unbalanced of creates slowness for Psychiatry to evolve scientifically.

The paper will present some strengths and weaknesses of DSM/ICD, RDoC, and Descriptive Psychopathology in Psychiatry. It will also discuss, with examples, the results of some relevant studies that can bring awareness of advances in research in Psychiatry. It will conclude with the author's suggestion on the steps to integrate DSM/ICD, RDoC, and Descriptive Psychopathology in teaching and practice.

## Methods

### Search methods for identification of papers:

The author searched the two following electronic databases: Google scholars and Pubmed from 1980 to 2018 for references including papers on DSM, ICD, Descriptive Psychology, and RDoC by free-texting: “comparison, strengths and weaknesses, Diagnostic and Statistical Manual of Mental Disorders, Research Domain Criteria, and Descriptive Psychopathology.”

### Criteria for considering studies:

#### Inclusion:

Regardless of the type of documents, the author included books, peer-review articles, comments, and editorials related to the strengths and weaknesses of DSM ICD, Descriptive Psychology, and RDoC. The authors then broadened the search to any document that compared DSM and ICD, RDoC and DSM, Descriptive Psychopathology, and DSM.

#### Exclusion:

**Figure 1:**
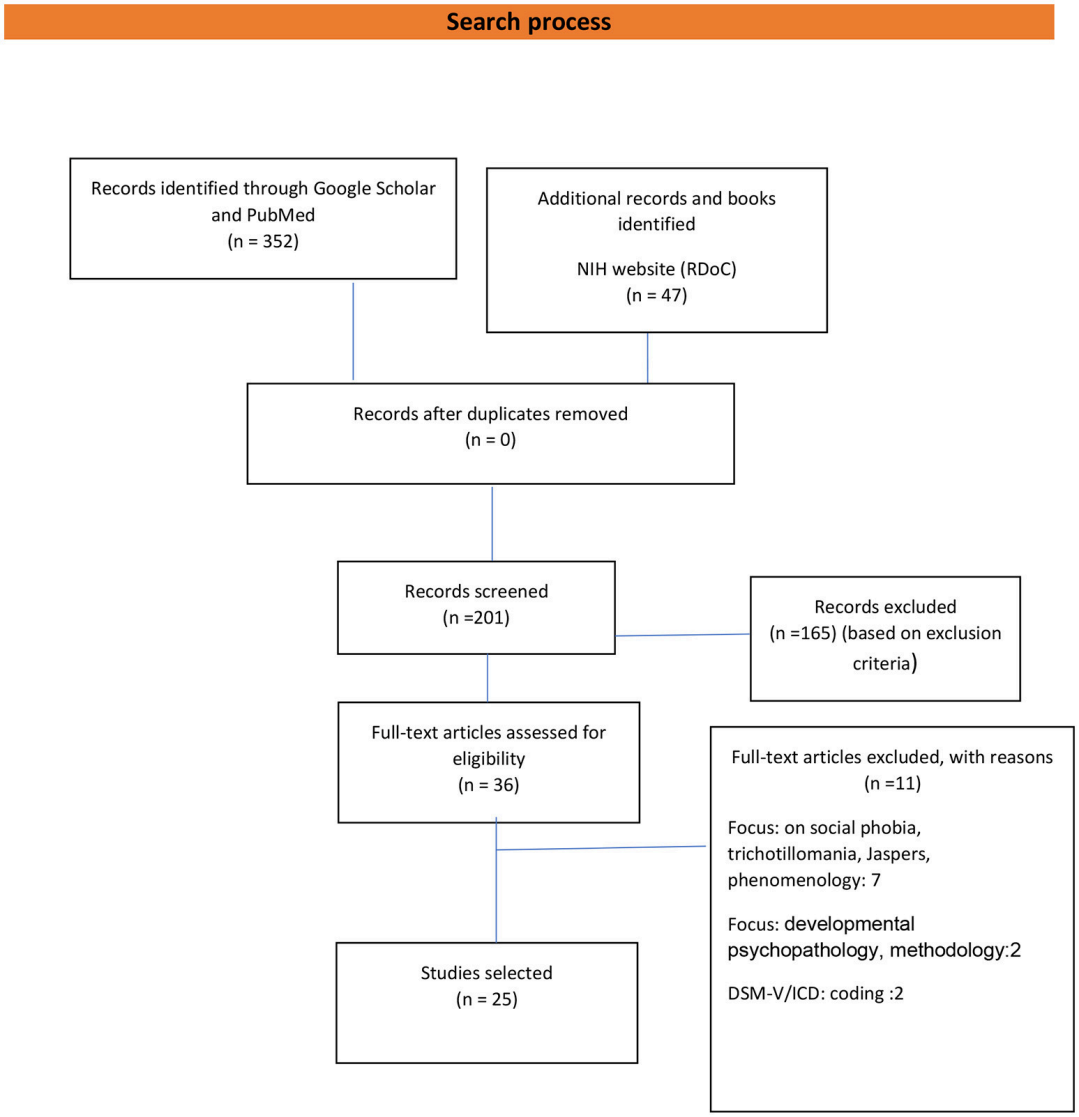
Flow diagram of the number of studies included and excluded. Adapted from Prisma: Moher et al. (1).

Papers on history, phenomenology, developmental psychopathology, coding or documents that focused on specific mental disturbance or behavior (Figure 1).

### Data collection and analysis:

The author extracted the following data: publication status, abstracts, type of analysis or critics, title, authors, names, source, country, and year of publication.

## Results

The initial electronic search originated 352 documents, and 47 additional papers were identified on the NIH website. An overall of 201 papers was screened for eligibility, and 36 texts were eligible for the paper (excluding 165 based on exclusion criteria). Of 36 documents, 25 of them were selected for the paper after excluding 11 papers with reasons (7 papers on social psychopathology, phobia, phenomenology, 2 on methodology, and 2 on coding) (Figure 1).

### Strengths and weaknesses

#### Diagnostic and statistical manual of mental disorders and the international classification of diseases (ICD)

In 1952, a new classification system of mental illness was released in the United States of America, the first edition of the DSM. For some, the DSM propels Psychiatry from a philosophical and heretical view to a critical thinking level. There are claims that the DSM is a source of references beyond billing purpose. In the United States of America, the DSM is recognized as a standard system for teaching, diagnosing and researching mental illnesses. Jonathan et al. analyzed the validity and reliability of the DSM-III criteria for posttraumatic stress disorder, the author found acceptable that interrater and test-retest reliabilities and diagnostic validity correlated to a standard diagnostic interview procedure (2). However, despite many changes over the year, the DSM contents have been criticized not to be able to conceptualize patient's constellation of symptoms (3, 4) accurately. Warelow et al. mentioned that the DSM-IV-TR diagnostic groups overlap, and their utility questionable. The author also suggested that some of the “conditions” have no clinical equivalents in practice (5). More drastically, it is reported that the DSM enhances research but limits the advancement of Psychiatry by generating additional unnecessary investigations, and some diagnoses may be unreal (6, 7). ICD is considered to be the international standard diagnostic tool for epidemiology, health management, and clinical purposes (8). Bowman mentioned that ICD-10 might be able to provide better data for evaluating and improving the quality of patient care and clinical research (9). Tyrer et al. stood behind a different point of view; that ICD was not able to stimulate research at the same level as the DSM (7). Overall, Hengartner et al. wrote that DSM and ICD do not appraise psychopathology in a manner that is satisfactory (10) (Table 1).

**Table 1:** Summary of the main findings/suggestion or perspectives of studies/documents/perspectives selected as well as comments to clarify the idea that drives the necessity of integrating DSM/ICD, Descriptive Psychopathology and RDoC in practice and teaching of psychiatry.

**References**	**Study/blog**	**Findings/perspective**	**Comments**
Berridge et al. (11)	Role of dopamine in reward	Dopamine systems necessary for ‘wanting'	Anhedonia as a symptom might not only be
		incentives, but not for ‘liking'	explained by the dopamine.
Berrios et al.(12), [ p. 12]	The history of mental symptoms	Mental symptoms; complex reflexions of	Clinian should be able to distinguish symptoms
		dysfunctional brain sites	that are noises or the one describing biological signals
Gordon (13)^*^	RDoC: Outcomes to Causes and Back	To establish statistical approach—Bayesian causal modeling#	DSM knows the outcomes(observations) not the
			causes (underlying disease processes)
Hengartner et al. (10)	Outline the major limitations	DSM/ICD does not define distinct natural entities	Genetic research and the neurosciences failed to aid
	of categorical psychiatric diagnoses	DSM/ICD diagnoses are not exclusive or exhaustive	psychiatric diagnoses or prognoses due to imposed limitations of
			DSM and ICD
Insel 2013^*^(14)	Transforming Diagnosis	DSM diagnoses are based on clusters of clinical symptoms	RDoC gears toward precision medicine to
		DSM diagnosis lack validity	diagnose and treat mental disorder
		Research Domain Criteria (RDoC) will incorporate genetics,	
		imaging, cognitive science for a new classification	
Kendell et al. (15)	Examine the evidence of Zone of rarity	No appropriate statistical techniques,	Author concluded there is little evidence that
	of mental disorders. Validity and	or clinical research strategies to determine whether there is	current psychiatric diagnoses are valid. However, does not
	utility of psychiatric diagnoses	Zone of rarity for mental disorder	exclude their usefulness
Kirk et al. (4)	Reliability of DSM	No credible evidence that DSM increases	The critic targeted DSM-III
		reliability	
Siegle et al. (16)	Time course of emotional	Depressed individuals show atypically sustained	Suggests that depression is a disorder
	information processing in	processing on emotional information processing tasks	characterized by sustained processing,
	depressed and nondepressed	(sustained pupil dilation)	a psychophysiological basis
Taylor et al. (3)[p. 5]	Beyond the DSM and ICD	Reduction of teaching the mental status examination	For Taylor, DSM/ICD approach patient care as a treatment mode
		and descriptive psychopathology	with rapid diagnosis and pharmacotherapy
		A skeletal view of psychopathology	
Treadway et al. (17)	Anhedonia in depression	Anhedonia in depression:	Anhedonia needs characterization, both
	Decisional anhedonia	positively-valence affective stimuli	clinical presentation and biological basis
		biological studies:	
		Motivation and decision-making	
Tyrer et al. (7)	Comparison of DSM and ICD	DSM promotes research	ICD is more comprehensive than DSM
		ICD better descriptions and definitions of disease	DSM is more accurate than ICD
Walter et al. (18)	Biological psychiatry, third wave	Mental disorders are brain disorders	Biological psychiatry has to approach theories of the mental
			constitution and insights of philosophy of mind (arts and science)
Woody et al. (19)	Integrating RDoC into depression	Depression shares 2 RDoC domains	An integration of depression risk into cognitive, genetic, and neural models
	research	Negative; Loss construct. Positive: Reward constructs.	

*The thought behind the integration is multifaceted: from the origin of psychiatry as philosophy (Walter et al.), categorical diagnosis checklist (Taylor et al., Hengartner et al., Kirk et al.) to the integration of genetics, biology, immunology, physiology and experimental psychology (Woody et al.)*.

* *Note that regarding Gordon 2017 and Insel 2013 they are online access perspectives not published articles or book references*.

# *Probabilistic relationships between diseases and symptoms*.

#### Descriptive psychopathology

Around (1910–1913), Karl Jaspers built the foundation for Descriptive Psychopathology; a comprehensive methodological arsenal for psychiatry (20). Descriptive Psychopathology assists in gathering information on a psychiatric condition (past and present history) (21), a precondition for a diagnostic (20). It collects information on specific signs and symptoms communicated by the patient, or a relative, and on what is observed by the examiner in an objective and precise manner. It facilitates better inter-rater reliability to diagnose mental illness (22). It permits a skilled examiner to observe abnormal behavior and subjective experience of a patient and interprets them as clinical signs or symptoms of brain dysfunction. Taylor et al. advocated that Descriptive Psychopathology understands that a “symptom” can be dubious. For example; “avolition,” a diminution of one motivation to initiate and perform self-directed purposeful activities can be a frontal syndrome or a depressive illness; “disinhibition” can be a frontal syndrome or mania (3). Häfner et al. wrote that the understanding of Psychology eases the understanding of the sick person, as well as the person biographical and personal context of the mental disorder (20).

Despite its strengths, Descriptive Psychopathology has been criticized for not possessing standard measure to appreciate with certainty the reliability of symptoms or signs (20). Ramos-Gorostiza et al. noted that Psychiatry has not been unable to compete with other fields of medicine or substitute “clinical semiotics” (merely descriptive clinical procedure) with a causal theory (23). A 2002 residency survey revealed there was a significant teaching issue of Descriptive Psychopathology in the United States of America. From an overall of (*n* = 149) residency programs, 47.1% of them did not offer a formal course in the use of DSM. Only 30% of the participants (*n* = 45) stated they were taught to recognize catatonic signs, extracampine or elementary hallucinations, oneiroid syndrome, Capgras, or Fregoli syndrome (3) (Table 1).

#### Research domain criteria

In 2008 the National Institutes of Health (NIH) launched RDoC as an initiative of evidence-based medicine to target and identify phenotypes and psychophysiological variables and psychobiological markers for mental disorders (24, 25). It includes psychological constructs or concepts, human behavior and functioning, and units of analysis (molecular, genetic, neurocircuit) [32]. The intention behind RDoC seems to approach mental illnesses with the Bayesian causal modeling (13, 26). This model will assist in finding relationships between causes and outcomes and vice versa. One of the major limitations of RDoC is the utilization of animal model that seems to be its backbone of RDoC. Some authors question the capability of RDoC to study schizophrenia, because, schizophrenia does not naturally occur in animals (24). Translational research has been slowly moving to human trials in every aspect of science due to several reasons (ethics, feasibility, setting, and regulations). Also, the idea of self-report is inconsistent with the RDoC framework (24).

### Example of some studies

#### Descriptive psychopathology; beyond compiling symptoms

This following example may elucidate a point of view. Taylor et al. (3) described a 51-year-old man who experienced substantial loss of interest and anhedonia; his mood was reactive, subdued, with mild diminished emotional expression rather than sadness or apprehension (3). On neurological examination, the patient presented slowness of movement and thought. The symptoms began after his trailer burned. Although the patient showed criteria for a major depressive disorder (insomnia, psychomotor retardation, trouble concentrating, anhedonia, no energy, not wanted to live), further investigation with a brain scan showed bilateral basal ganglia calcification consistent with carbon monoxide exposure. Methylphenidate treatment improved the patient condition (3). A symptom can be disparate, and it is critical to elucidate the neurobiological pathways involving that symptom. In the case of anhedonia, for example, a dissection into motivational and hedonic aspects of anhedonia is essential (17). This approach will help us decode probable circuits of anhedonia. Treadway et al. questioned the explanation of anhedonia via the dopamine hypothesis. The author argued that such an approach might be the reason for the failure to dissociate consummatory and motivational aspects of reward behavior (17). Treadway suggested that we need to move away from the concept of anhedonia as mood-like phenomena. Studies in rats found that 6-hydroxydopamine (6-OHDA) lesions of nucleus accumbens (NAcc DA) synapses do not impair hedonic liking expressions of the rodents (11). It seems to be essential to approach a “symptom” with a deeper understanding. For example, one can broaden anhedonia, a pivotal element to diagnose depression, as a deficit of the hedonic capacity (“liking”) and motivation and reinforcement (“wanting”). As one can realize, this new way to comprehend anhedonia veers our thinking to a variety of brain regions, mesolimbic and mesocortical neural circuits, and neurotransmitters; dopamine, glutamine, and gamma-aminobutyric acid. Treadway concluded that if there is an improvement in the assessment of “motivational anhedonia,” it might help in predicting treatment response (17).

#### The need for RDoC

Psychiatry is evolving, and there is more to know and understand. Considering schizophrenia as a diagnosis, around 80% of its variance is genetic, and there is no single gene for the disorder (18, 27). Woody et al. reviewed depression within the RDoC framework to address the “heterogeneity” of depression based on the “Loss construct.” Based on the Loss construct explanation; depression might be associated with a “Circuit disruption.” The cortico-limbic circuitry is disrupted and overreacted to surrounding stimuli. There is a reduction of activity in the prefrontal cortex as well as diminished functionality between regions, and augmentation of events in the default mode network (19). Genetically, some genes regulate the neurotransmission of monoamines (5-HTTLPR, 5-HT receptor genes, MAOA, and COMT) (19). At the molecular level; glucocorticoids, sex hormones (estrogen and androgen), oxytocin, vasopressin, and cytokines derangement cause depression. Physiologically, the autonomic nervous system (ANS), hypothalamic-pituitary-adrenal (HPA) axis, and neuroimmune are dysregulated. There is pupil dilation (16, 19). The behavior, cognition, and emotion are impaired creating sadness, anhedonia, guilt, morbid thoughts, psychomotor retardation, and deficits in executive function, and disruptions in sleep, appetite, and libido, as well as rumination and biases in attention and memory (19).

Psychiatry holds research data that can help the field to emulate others like Neurology, Internal Medicine, etc. Psychiatry's scientific knowledge appears to be spread between DSM/ICD, Descriptive Psychopathology, and RDoC, thus integrating this knowledge in teaching and practice seems to be imperative for the advancement of this branch of medicine (Table 1).

## Discussion

Kendell et al. questioned whether psychiatric syndromes are separated from one another by the “zone of rarity.” Snealth called a “point of rarity” the possibility that disorders might cluster into one another where there is no natural boundary (14). The same way Kendell et al. coined the concept of “zone of rarity” which can be defined as a lacuna(hiatus) that exists between the hallmark(features) of a biological disorder with a definite diagnosis and other conditions that do not carry this diagnosis (7, 15).

Tyrer et al. exemplified the zone of rarity in a diagram of depression and three causes of anemia (7). For Tyrer, a blood analysis can differentiate pernicious anemia, iron deficiency anemia or lymphocytic leukemia which is not the case for a major depressive disorder (MDD) because of the lack of a zone of rarity. Psychiatry does not possess specific markers to diagnose depression. Kendell added that the main problem is not of a demonstration of no zone of rarity between diagnostic categories, or not having statistical techniques to determine boundaries that exist for a mental disorder, but research has not been done yet to elucidate this problem (15).

Let us analyze the assessment of an Internist examining a patient for a complaint of a cough. A cough, a symptom, can be anything from a common cold to pneumonia. However, the Internist does not simplistically label a cough as pneumonia. Also, besides improving the cough, the internist will also manage symptoms affecting the patient well-being (fever). Each symptom accompanied the illness can present a specific pathology, a specific biological explanation, and might need a treatment approach even the patient is on antibiotics for pneumonia. The author believes that psychiatric diagnosis may have a “zone of rarity.” Some markers for schizophrenia were discovered, but they may not be specific of the illness. A patient taking a selective serotonin reuptake inhibitor (SSRI) for depression can receive a different treatment to alleviate his or her anhedonia. Anhedonia itself can involve different anatomical sites or neurotransmitters. One can target the primary symptom and probably achieve better results besides prescribing an SSRI as the magic wand. Such attention to detail needs to be in the mind of the examiner. Otherwise, the patient will be a diagnosis and be treated as such.

The author compared side by side the Harrison's Internal Medicine (28) write up on pneumonia to Sadock and Kaplan's Comprehensive Text of Psychiatry (29) of a mood disorder like depression (Table 2). The chapter on depression from Sadock does not profile precisely the way pneumonia is presented in Harrison's, but there is enough data to write depression the same way Harrison's did. One can argue that Psychiatrists do not need to model other fields, but some also might argue that adopting other's model of writing may facilitate better communication between peers. A psychiatric interview might represent a verbatim from the patients like “I feel tired most mornings” (31) which is a Hermeneutical approach of humanities (theory and methodology of interpretation rooted in the interpretation of biblical texts, wisdom literature, and philosophical texts) (32). Also, “I feel tired most mornings” conceptualized an instrumentalist approach of Scientific Empiricism—the practice of relying on observation and experiment especially in the Natural Sciences (33)—which contrasts with Neuroscience that uses assumptions based on Scientific Realism (recommending belief in both observable and unobservable aspects of the world described by the sciences) (33). Thus, in place of “I feel tired most mornings,” “I feel restless” may represent an occurrence state which might better translate to an imaging data gaining a clinical meaning (31). Psychiatry possesses tools with high sensitivity, specificity, and reliability to monitor treatment responses. Psychiatry is symptom-driven not because of a lack of substantial research to better treat or diagnose mental illnesses, but because of poor utilization of scientific data, and a lack of application of reliable information in practice and teaching.

**Table 2:** The chapter on mood disorder from Sadock's is not precisely like the write up on pneumonia from Harrison's.

	**Pneumonia (Harrison's)**	**Mood disorders: major depressive disorder (Sadock's)**	
Definition	Pneumonia is an infection of the pulmonary parenchyma	A large group of psychiatric disorders in which pathological moods and related vegetative and psychomotor disturbances dominate the clinical picture
Pathophysiology	Pneumonia results from the proliferation of microbial pathogens at the alveolar level and the host's response to those pathogens	Disturbances in all four spheres: mood, psychomotor activity, cognitive, and vegetative
Pathology	Initial phase is edema	Immunological Disturbance: Structural and Functional Brain dysfunction: Alterations of Sleep Neurophysiology: Thyroid Axis Activity: Genes:	decreased lymphocyte proliferation hyperintensities in subcortical regions premature loss of deep (slow wave) sleep blunted TSH^*^ response to TRH^*^ challenge solute carrier family 6 member 3, dopamine receptor D4
Etiology	Bacteria, fungi, viruses, and protozoa	Acute stress responses involve activation of central and peripheral components of two interactive psychoneuroendocrine systems Physical, verbal, and sexual abuse and parental neglect Substance abuse
Epidemiology	More than 5 million CAPS^*^ cases occur annually in the United States	Lifetime prevalence estimates average 11.1 (range 8.0 to 18.4) in low and 14.6 (range 6.6 to 21.0) in high-income countries
Clinical manifestations	Febrile with tachycardia and Cough	Markedly diminished interest or pleasure in all(anhedonia)	
Physical examination:	Tactile fremitus	Psychomotor Disturbances Fold often associated with depression: Veraguth's fold
		DSM-V criteria:	1-Depressed mood most of the day 2.Markedly diminished interest or pleasure in all 3-Significant weight loss 4-Insomnia or hypersomnia 5.Psychomotor agitation or retardation
Diagnosis Clinical Diagnosis: Infectious Noninfectious			
			Patient Health Questionnaire-9 (PHQ-9) Beck Depression Inventory (BDI) Thyroid-stimulating hormone (TSH) Blood and urine toxicology screen Complete blood cell (CBC) count Blood alcohol level
Etiologic Diagnosis	Gram's Stain and Culture of Sputum		
Treatment	Antibiotic managment	Serotonin reuptake inhibitor, electroconvulsive therapy
Complications:	Respiratory failure		Suicide. Impairment of social functioning. Cardiovascular, metabolic syndrome

Adapted from Kaplan and Sadock (30). Thyroid Stimulating Hormone^*^ (TSH).

Thyroid Releasing Hormone^*^ (TRH). Community -Acquired Pneumonia: CAP^*^.

## Conclusion

Despite extensive research and findings within Neuroscience, Genetics, Cellular Molecular Psychiatry as well as studies in Descriptive Psychopathology, the teaching and practice of Psychiatry does not fully integrate these side by side advances with the DSM, especially in the US. Very often, Psychiatrists describe symptoms as a layperson lacking clear imprints. There are some stipulations that Psychiatrists do not exploit evidence-based information and data (standardized self-reports, clinician-administered scales, genetics or neuroimaging findings) available to them to diagnose or treat patients with mental illness.

### Author's perspectives for the integration

**Figure 2:**
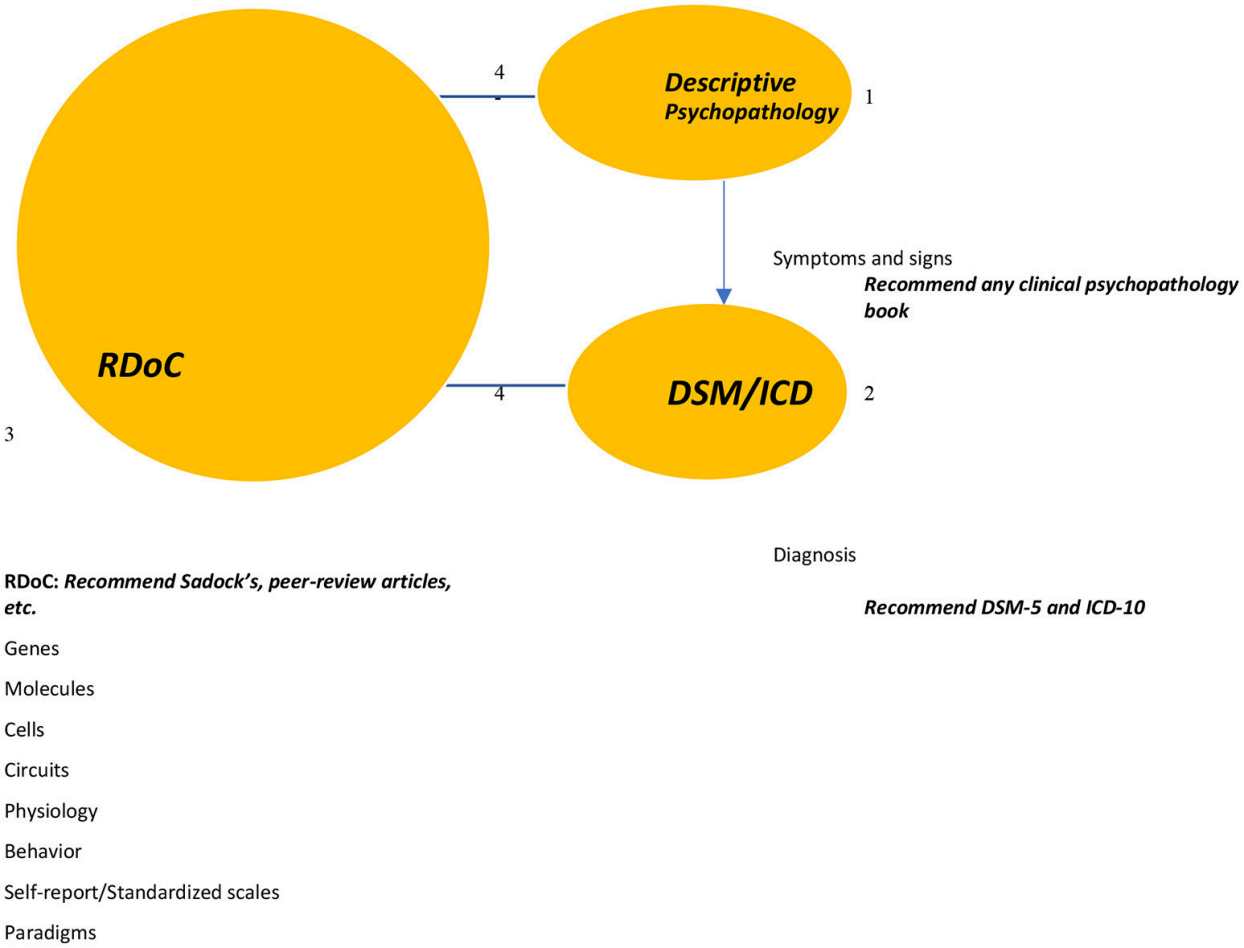
This diagram illustrates the concept of the integration. 1-The idea is to start the patient assessment by dissecting and analyzing his or her symptomatology. 2-An attempt will be made to fit the patient symptoms and signs into a diagnosis. 3-The third step is to attempt to understand the patient symptoms, probable DSM diagnosis (regardless a diagnosis) by using scientific data (markers, imaging, molecules, expected behavior, scores on standardized scales. 4-The process is dynamic between RDoC and DSM/ICD/ Descriptive Psychopathology. Adapted from Prisma: Moher et al. (1).

The author suggests that the DSM and ICD remain both a guide for diagnosis, billing, and coding purposes. The principles of teaching or practicing of Psychiatry should start with an in-depth analysis and understanding of specific symptoms and signs (dissecting the mind) during the psychiatric evaluation. After data are appropriately collected, one will attempt to elaborate a differential diagnosis that might fit the DSM/ICD. This attempt must not rely on the number of symptoms or a checklist. The author predicts that it will be sometimes difficult to fit symptoms within a DSM or ICD approach. In that case, RDoC can be an excellent tool as the next attempt to match data collected to be able to broaden the assessment to treat the patient. RDoC can also assist in hypothesizing on the probable etiologies of a presenting mental disorder (Refer to RDoC Snapshot: Version 4 (saved 5/30/18) at https://www.nimh.nih.gov/research-priorities/rdoc/constructs/rdoc-snapshot-version-4-saved-5-30-18.shtml). This idea does not imply that one needs to wait and keep searching for an underlying cause of a presenting mental illness before treatment starts, but the treatment plan should be based on sound scientific hypotheses. The idea of assessing a patient with preset DSM/ICD criteria is discouraged and that DSM/ICD diagnosis should not be considered as the sole and only hypothesis to diagnose or treat mental illness (Figure 2, illustrative diagram). However, the integration should be dynamic in a way to avoid calculating symptoms to fit a diagnostic criterion in the case of the DSM, lingering too much in heretical manifestations in the case of the Descriptive Psychopathology or thinking of the patient as a lab or an imaging result in the case of RDoC.

### The gain

The integration will enhance the development of sound and scientific theoretical frameworks of etiology, epidemiology, and pathology of mental illnesses, and diagnostic reliability. It will improve communication between psychiatrists and their peers. Lastly, it will assist in targeting better psychiatric medications.

## Author contributions

The author confirms being the sole contributor of this work and has approved it for publication.

### Conflict of interest statement

The author declares that the research was conducted in the absence of any commercial or financial relationships that could be construed as a potential conflict of interest.
